# Chemical mediation of coral larval settlement by crustose coralline algae

**DOI:** 10.1038/srep10803

**Published:** 2015-06-04

**Authors:** J. Tebben, C. A Motti, Nahshon Siboni, D. M. Tapiolas, A. P. Negri, P. J. Schupp, Makoto Kitamura, Masayuki Hatta, P. D. Steinberg, T. Harder

**Affiliations:** 1 School of Biological Earth and Environmental Sciences, Centre for Marine Bio-Innovation, The University of New South Wales, Sydney, 2052 NSW, Australia; 2 Australian Institute of Marine Science, Townsville, 4810 QLD, Australia; 3 Plant Functional Biology and Climate Change, University of Technology Sydney, Ultimo, 2007 NSW, Australia; 4 Institute for Chemistry and Biology of the Marine Environment Terramare, University of Oldenburg, 26382 Wilhelmshaven, Germany; 5 Okinawa Environment Science Center, Urasoe, Okinawa 901-2111, Japan; 6 Graduate School of Humanities and Sciences, Ochanomizu University, Tokyo 112-8610, Japan; 7 Sydney Institute of Marine Science, Mosman, 2088 NSW, Australia; 8 Advanced Environmental Biotechnology Centre, Nanyang Technological University, Singapore 639798, Singapore

## Abstract

The majority of marine invertebrates produce dispersive larvae which, in order to complete their life cycles, must attach and metamorphose into benthic forms. This process, collectively referred to as settlement, is often guided by habitat-specific cues. While the sources of such cues are well known, the links between their biological activity, chemical identity, presence and quantification *in situ* are largely missing. Previous work on coral larval settlement *in vitro* has shown widespread induction by crustose coralline algae (CCA) and in particular their associated bacteria. However, we found that bacterial biofilms on CCA did not initiate ecologically realistic settlement responses in larvae of 11 hard coral species from Australia, Guam, Singapore and Japan. We instead found that algal chemical cues induce identical behavioral responses of larvae as per live CCA. We identified two classes of CCA cell wall-associated compounds – glycoglycerolipids and polysaccharides – as the main constituents of settlement inducing fractions. These algae-derived fractions induce settlement and metamorphosis at equivalent concentrations as present in CCA, both in small scale laboratory assays and under flow-through conditions, suggesting their ability to act in an ecologically relevant fashion to steer larval settlement of corals. Both compound classes were readily detected in natural samples.

One common reproductive strategy of marine invertebrates is via dispersive, planktonic larvae which must recruit onto appropriate benthic surfaces to complete their life cycle. This transition involves larval attachment to surfaces and is accompanied by rapid and drastic morphological changes as planulae larvae metamorphose into benthic polyps. These attachment and transformation events, referred to as “larval settlement and metamorphosis”, are coordinated by complex and often poorly understood exogenous and endogenous factors. While exogenous factors capable of inducing larval settlement – commonly referred to as settlement cues - are diverse, ranging from environmental parameters to chemical extracts or even purified compounds, they all convey information about the post-settlement habitat^1^. Larval settlement and metamorphosis in response to these cues is often highly selective, presumably because of the detrimental consequences of recruitment in sub-optimal habitats^2^. In the presence of certain chemical compounds, coral larvae are also observed to metamorphose without attachment in laboratory assays^3^,^4^. Notably, in the absence of settlement cues, many marine invertebrate propagules delay or cease settlement altogether^5^,^6^, indicating a crucial role for these cues in marine benthic communities.

Common natural sources of larval settlement cues include microbial biofilms, prey and predator odours, conspecifics and algae. However, our understanding of the chemical ecology of surface colonization in marine benthic systems is far less advanced than studies of chemically mediated plant-herbivore or predator-prey interactions, or indeed of deterrents of larval settlement^7^. This is because, unlike these other interactions, few inducers of invertebrate larval settlement have been chemically characterized, quantified *in situ* and shown to induce settlement at naturally occurring concentrations^8^. This paucity of information is a critical gap in our understanding of chemical mediation of surface colonisation in the marine environment and is addressed here for corals, the major foundation species of coral reefs.

While physical environmental parameters associated with reef surfaces, such as topography, color and sound have been shown to affect coral larval settlement^9^,^10^,^11^, the most commonly identified and effective sources of larval settlement and metamorphosis inducers are biological: crustose coralline algae (CCA) and reefal biofilms. Specific bacterial isolates from reefal biofilms have been shown to alter larval behavior in laboratory assays, particularly those within or affiliated with the genus *Pseudoalteromonas*^3^,^4^,^12^,^13^, and in two independent laboratory studies the causative bacterial metabolite was identified as tetrabromopyrrole (TBP)^4^,^12^.

In the case of Caribbean coral species, TBP induced comparatively high levels of settlement and metamorphosis, leading to the proposition that TBP was a compound of widespread ecological importance for larval settlement^12^. However, this and our previous studies observed that TBP and TBP-producing bacteria triggered variable degrees of larval settlement and metamorphosis and larval metamorphosis without attachment^4^,^12^, i.e. larvae often did not accomplish the complete transition to an attached, metamorphosed benthic juvenile.

We therefore further investigated the ecological plausibility of TBP as a coral larval settlement cue. In a broadly scaled study, including several coral species across the Indian and Pacific Ocean, we found ample evidence that TBP and TBP-producing bacteria commonly caused high levels of larval metamorphosis without attachment. This unusual response, in which exogenous signals induce only a subset of the recruitment chain in a large portion of larvae, is well known from other marine invertebrates (hydroids, oysters) where juvenile development without attachment can be triggered with hormonal signalling compounds^14^,^15^,^16^,^17^,^18^ , which are not kown to be present *in situ*. The discrepancy in larval behaviour to TBP versus CCA was the main reason to experimentally address if TBP-producing bacteria occured at sufficiently high concentrations *in situ* to evoke any ecologically meaningful outcome on coral larval settlement.

An alternative to biofilm-derived cues for coral larval settlement and metamorphosis are those derived from the CCA themselves. In pioneering work, Morse and Morse enzymatically liberated an insoluble settlement and metamorphosis cue for agaricid corals from decalcified CCA^19^. This morphogen was suggested to be associated with or to contain a sulfated glycosaminoglycan, but was not structurally elucidated and thus its role as inducer of acroporid coral larval settlement remained unclear. Simple extraction of CCA with alcohols has since been demonstrated to yield extracts that contain potent larval settlement and metamorphosis cues for many hard corals^20^,^21^,^22^,^23^. However, the demonstration that the cues in these extracts mediate larval settlement *in situ* has remained largely unexplored, with the notable exception of Raimondi and Morse^4^.

Here we elucidated the diversity and efficacy of CCA-associated cues affecting coral larval behavior and assessed their potential to act as ecologically plausible cues for coral larval settlement and metamorphosis. Both bacterially-derived and algal-derived cues from CCA were characterized and quantified where possible, and the behavior of coral larvae to these cues compared with the response to CCA in both laboratory scale assays and in mesocosms under flow-through conditions. Moreover, in order to explore the generality of these cues, this study was conducted with corals from a wide geographic area, across the Pacific and the Indian Oceans (Australia, Singapore, Guam, Japan), and with corals from different taxa (broadcast spawning acroporidae, siderastreidae, favidae and a brooding favidae).

## Results

### Larval bioassay-guided screening of bacterial isolates

Strain AKA07-7a (AB571947), one of 160 bacterial isolates from Okinawa, induced high levels of coral larval metamorphosis without attachment. This strain was identified as a close affiliate (99% sequence similarity of the 16S rRNA gene) to *Pseudoalteromonas* strains A3^3^, J010^4^ and PS5^12^. The bioassay-guided fractionation of this strain’s crude extract yielded TBP as the only metabolite with an effect on coral larval metamorphosis (as per^4^).

### Larval response to TBP

**Figure 1:**
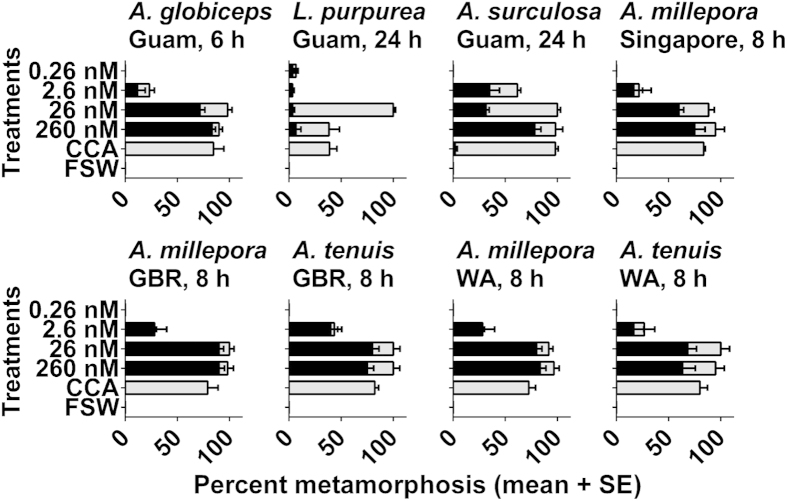
Larval response (+SE, n = 6 – 10) to a serial dilution of TBP after 6, 8 or 24 h. Five coral species (*Acropora millepora, A. tenuis, A. globiceps, A. surculosa,* and *Leptastrea purpurea*) from four locations (Great Barrier Reef (GBR), Ningaloo Reef (WA), Guam and Singapore) were used in larval settlement assays with TBP together with controls of filtered seawater (FSW) and live crustose coralline algae (CCA). *A. humilis, A. intermedia, A. digitifera, A. tenuis, Montipora hispida, Favia pallida* and *Pseudosiderastrea tayamai* (Okinawa) were tested with TBP purified from bacterial extracts (see Supplementary Fig. S1 online). Black bars: mean percentage of larval metamorphosis without attachment, Grey bars: mean percentage of larval settlement and metamorphosis (with attachment).

In assays performed in the GBR, WA, Singapore and Guam, TBP (synthesized or isolated from *Pseudoalteromonas* AKA07-7a) significantly induced larval settlement and metamorphosis of *Acropora millepora, A. tenuis, A. globiceps, A. surculosa,* and *Leptastrea purpurea* (p < 0.01, Fig. 1) compared to the 0.2 μm filtered seawater (FSW) control. TPB also induced significant levels of metamorphosis without attachment in all coral larval species except for *L. purpurea* (p = 0.57). Notably, none of these coral species exhibited larval metamorphosis without attachment in response to live CCA. Instead, in the presence of live CCA, all species showed significant levels of larval settlement and metamorphosis (Fig. 1) consistent with previous reports^3^,^4^,^20^. Overall, only a single larva out of 660 (*A. surculosa*) exposed to live CCA metamorphosed but did not attach (Fig. 1). In contrast, more than 300 larvae (54% average) across the different coral species tested metamorphosed but failed to attach in response to TBP at the highest concentration tested.

Where available, corals were tested with TBP extracted and purified from locally obtained bacterial isolates. There was no significant difference in the larval response of *A. tenuis* and *A. millepora* (GBR) to bacterially-derived (purified from *Pseudoalteromonas* J010) and synthetic TBP. For assays performed in Okinawa, TBP isolated from *Pseudoalteromonas* strain AKA07-7a significantly induced larval settlement and metamorphosis of *A. tenuis, A. humilis, A. intermedia, A. digitifera, Montipora hispida, Favia pallida* and *Pseudosiderastrea tayamai* (p < 0.01). TPB also induced significant levels of larval metamorphosis without attachment in all coral species except for *F. pallida* (p = 0.39).

### Response of pre-competent coral larvae to TBP

**Figure 2:**
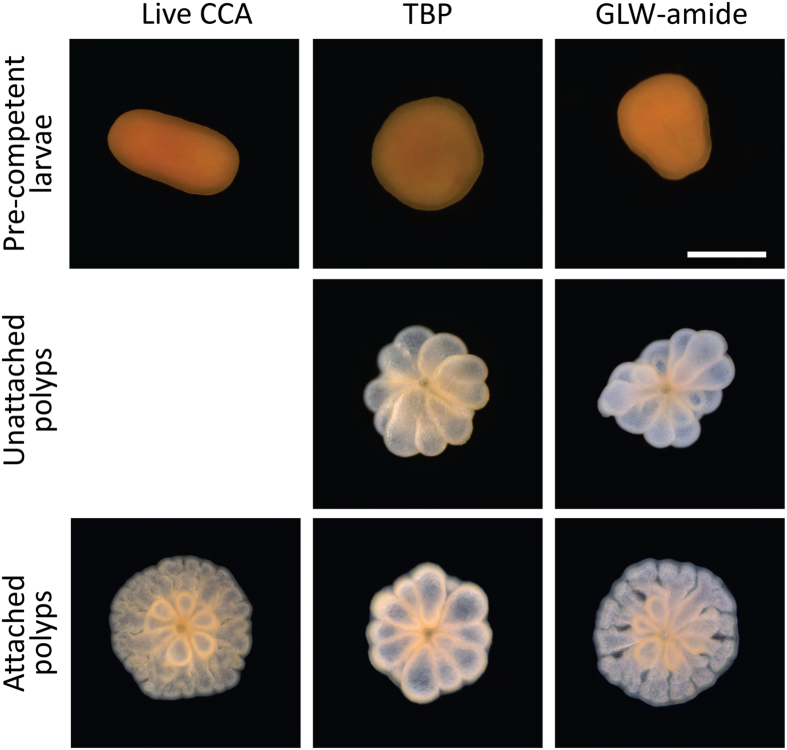
Photographs of *Acropora tenuis* larvae and juveniles exposed to different cues. Pre-competent larvae (2 day-old, top row) and competent larvae (6 day-old, middle and bottom rows) were exposed to live CCA, TBP and GLW-amide. Images were taken 24 h after exposure. Pictures show typical metamorphosed polyps (attached (bottom) and unattached (middle)). Scale bar = 500 μm.

The behavioral response of pre-competent larvae of *A. millepora* and *A. tenuis* to TBP was observed at 24 h intervals post spawning (1 to 4 days). During this time frame larvae were either significantly immobilized or died in the presence of TBP, whereas larvae were unaffected in the FSW control (*A. millepora*: p < 0.0001); *A. tenuis*: p < 0.0001). The pre-competent larvae were not adversely affected in the presence of live CCA and continued to develop normally (Fig. 2).

### Quantification of TBP-producing Pseudoalteromonads on the surface of CCA

The efficiency of all real-time quantitative PCR runs ranged between 0.96 – 1.0 (R^2^ ≥ 0.995). The correct size of q-PCR products was confirmed by electrophoresis (see Supplementary Material and Methods online). The abundance of total Pseudoalteromonads on the CCA *Porolithon onkodes* was 2.1 ± 1.2 mm^−2^ (mean cell number ± SE, Sept. 2010, Supplementary Table S1 online) and 1.3 ± 1.1 mm^−2^ (Dec. 2010). The total abundance of Pseudoalteromonads on the CCA *Neogoniolithon fosliei* was 10 ± 0.7 mm^−2^ (Sept. 2010) and 2.7 ± 2.1 mm^−2^ (Dec. 2010). The abundance of TBP-producing *Pseudoalteromonas* strains J010 and J021 on *P. onkodes* was 0.01 ± 0.007 mm^−2^ (September 2010) and 0.06 ± 0.03 mm^−2^ (Dec. 2010) and on *N. fosliei* 0.02 ± 0.005 mm^−2^ (Sept. 2010) and 0.002 ± 0.001 mm^−2^ (Dec. 2010).

### Quantification of TBP in bacterial and CCA samples

Electrospray mass spectrometry (ESI-MS) of TBP in negative mode produced the expected typical isotopic pattern of *m/z* 377.9, 379.9, 381.7, 383.6, 385.6 (1:4:6:4:1). The same diagnostic isotopic pattern was detected by LC-MS, ESI-FTMS and DESI-MS in 10^−5^ dilutions of the lowest metamorphosis-inducing concentration of TBP, and also in bacterial extracts. TBP could not be detected in settlement-inducing CCA extracts or chromatographic fractions of *P. onkodes*, *Hydrolithon reinboldii* and *N. fosliei.*

### Experimental treatments to release or inactivate larval settlement cues from CCA

**Figure 3:**
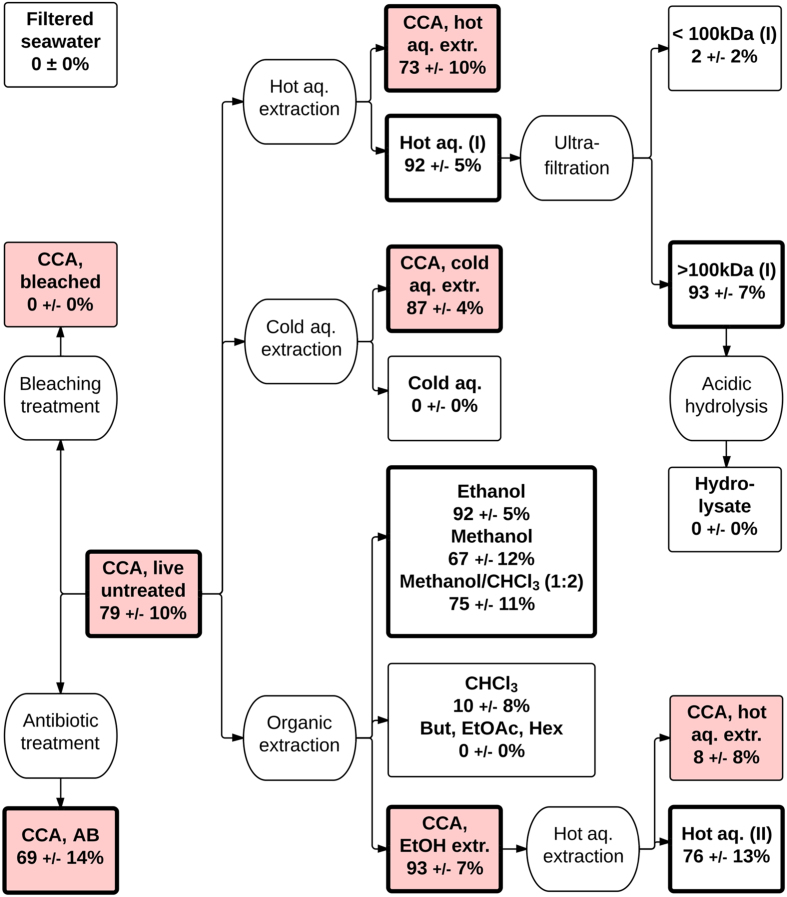
Experimental flowchart of bioassay-guided treatments to release or inactivate larval settlement cues from live CCA (*Hydrolithon onkodes*). Experimental treatments (ovals) and their resulting samples (rectangles) are shown. CCA particulate samples are highlighted in red, liquid extracts in white. Percentage values represent the mean larval settlement response (±SE, n = 6) of *Acropora millepora* to these samples after 12 h. Bold rectangles represent samples that (i) induced significant larval settlement and metamorphosis compared to the negative control of Filtered seawater (p < 0.05, pairwise PERMANOVA), and (ii) statistically the same response to live, untreated CCA (α = 0.05, pairwise PERMANOVA). Abbreviations: CHCl_3_ (chloroform), BuOH (butanol), MeOH (methanol), EtOH (ethanol), EtOAc (ethylacetate), Hex (hexane), aq. (aqueous), extr. (extracted), AB (antibiotic).

The larval response of *A. millepora* to live and treated CCA as well as different extract qualities is summarized in Fig. 3. The induction of larval settlement and metamorphosis by live, untreated CCA (79 ± 10% [mean ± SE ] was not affected by treatment of CCA with antibiotics (69 ± 14%), extraction with ethanol (93 ± 7%), hot aqueous extraction via autoclaving (73 ± 10%) or grinding followed by extraction with cold water (87 ± 4%). On the contrary, chemical bleaching (0 ± 0%) and extraction of CCA with ethanol followed by hot aqueous extraction (8 ± 8%) largely eliminated their potential to induce larval settlement and metamorphosis. The hot aqueous extraction of previously ethanol extracted CCA yielded an inductive aqueous extract (76 ± 13%). Of the extraction solvents employed ethanol (92 ± 5%), methanol (67 ± 12%), and a methanol:chloroform [1:2] mixture (75 ± 11%) yielded larval settlement and metamorphosis-inducing CCA extracts. The cold aqueous CCA extract (0 ± 0%) did not elicit a response whereas the hot aqueous CCA extract (92 ± 5%) induced high levels of settlement and metamorphosis. This hot extract was of high molecular weight given its retention on a 100 kDa filter membrane (93 ± 7%). The filtrate (<100 kDa) did not induce larval settlement (2 ± 2%). Subsequent total hydrolysis of the high molecular weight fraction rendered it inactive (0 ± 0%). All samples described above as ‘inductive’ resulted in significantly higher percentages of larval settlement and metamorphosis compared to the FSW control (p < 0.005) and the same percentage of larval settlement compared to the live CCA positive control (p > 0.05). In settlement assays with *A. tenius* larvae (data not shown), the same statistical distinction of inductive and non-inductive samples was observed as with larvae of *A. millepora*. The inductive properties of these samples were reproducible across multiple spawnings, several years (2010–2013) and different locations (GBR, WA and Singapore).

### Bioassay-guided fractionation of settlement-inducing algal extracts

The organic soluble settlement and metamorphosis cues present in ethanol, methanol and methanol:chloroform [1:2] extracts of *P. onkodes* were confined to two vacuum flash chromatography (C18-VFC) fractions (Fr.2, 92 ± 6% (mean settlement ± SE) and Fr.3, 94 ± 4%), two SiO_2_-VFC fractions (Fr.8, 77 ± 8% and Fr.9, 75 ± 5%), and two SiO_2_-TLC fractions (Fr.14, Mobile Phase A, R_*f*_ : 0.62, 90 ± 6% and Fr.17, Mobile Phase B, R_*f*_ : 0.20, 97 ± 3%). Fr.11 and Fr.20 represented chromatographically unresolved fractions containing the compounds present in Fr.17 and Fr.14, respectively (Supplementary Fig. S2). Larval settlement and metamorphosis of *A. millepora* and *A. tenuis* in response to all of these fractions was significantly higher than to the FSW control (p < 0.005) and did not differ from live CCA controls (α = 0.05). Both, Fr.14 and Fr.17 contained visible impurities, evident by a blue fluorescence visualized under λ 365 nm. To separate these co-eluting compounds Fr.14 and Fr.17 were re-chromatographed to yield spectroscopically (^1^H-NMR) pure compounds **1** and **2**. The concentration of separated impurities was below ^1^H-NMR detection levels. At this purification level, neither **1** or **2**, nor the pooled fractions recovered across the entire TLC-lane induced larval settlement at any of the concentrations tested. Given their strong ionization characteristics, these lipids are ideal mass-spectrometric markers to guide repeated isolation of settlement-inducing fractions from organic CCA extracts.

### Structural elucidation of **1** and **2**

The molecular formula of **1** was established as C_25_H_42_O_9_ based on ESI-FTMS (*m/z* [M + Na]^+^ 509.2724, calculated for C_25_H_42_O_9_Na^+^ 509.2721) and its structure confirmed by ^1^H- and ^13^C-NMR (Supplementary Table S2, Table S3) as (2*S*)-1-*O*-(7*Z*,10*Z*,13*Z*-hexadecatrienoyl)-3-*O*-β-D-galactopyranosyl-*sn*-glycerol (MGMG; Supplementary Fig. S2, A) . Similarly, the molecular formula of **2** was established as C_25_H_48_O_11_S (*m/z* [M – H + 2Na]^+^ 601.2608, calculated for C_25_H_47_O_11_SNa_2_^+^ 601.2629; [M – H]^-^ 555.2894, calculated for C_25_H_47_O_11_S^-^ 555.2845) and its structure as (2*R*)-1-*O*-(palmitoyl)-3-*O*-α-D-(6’-sulfoquinovosyl)-*sn*-glycerol (SQMG; Supplementary Fig. S2, B). Spectral data of both compounds were in agreement with literature values^20^,^21^,^22^,^23^,^24^.

### Larval settlement assays with immobilized CCA-derived cues in large water volumes under flow-through conditions

The petri-dish assay format widely used (including in this study) to screen larval settlement cues is prone to artifacts because larvae are exposed to cues in very small volumes of stagnant water, possibly confounding a real response of larvae for the cue surface. To determine larval responses in a more realistic setting, assays were also conducted in 500 L (n = 3) tanks of flowing seawater using the cues obtained by 100 kDa ultrafiltration of the hot aqueous CCA extract and VFC of CCA extracts (pooled fractions 8 and 9; see SI Materials and Methods). These cues were embedded in resins (n = 15, paraffin and carrageenan) at the equivalent volumetric concentration as in CCA. Resins containing the cues induced significantly more settlement and metamorphosis (p < 0.001, Tukey’s) than resins lacking embedded cues, for which settlement was not distinct from zero (Supplementary Fig. S4).

## Discussion

Both microbial biofilms and a variety of CCA species have been identified as inducers of larval settlement of hard corals^20^,^25^,^26^,^27^,^28^. Recently, a number of Pseudoalteromonads isolated from both natural and artificial reef surfaces have received significant attention in the literature for their ability to elicit variable degrees of both larval settlement and metamorphosis of corals and other marine invertebrates^3^,^4^,^12^,^13^,^29^. However, the presence of these bacteria and their putative settlement cue TBP in reef ecosystems at inductive concentrations has not been confirmed, thus the ecological relevance of these bacteria as natural inducers of larval settlement remains unclear.

In this study, we investigated the ecological plausibility of Pseudoalteromonads and TBP as a biofilm-derived larval settlement cue, which has been proposed as a compound of widespread ecological importance for coral larval settlement in the Caribbean^12^. Using multiple hard coral species, we found that TBP-producing Pseudoalteromonads obtained from epiphytic and epilithic biofilms did not elicit the same rates of coral larval settlement and metamorphosis as CCA, and in addition introduced morphogenic processes (metamorphosis without attachment) that are likely fatal to larvae. Having determined a lack of compelling evidence for a bacterial source of settlement cues, focus shifted to the CCA *per se*. Two monoacylated glycoglycerolipids and a partially characterized high molecular polysaccharide from *P. onkodes* were identified as the main components of purified fractions that caused larval settlement and metamorphosis as the CCA *per se* (Table 1).

In four separate studies across three geographic regions, the only bacterial isolates to elicit coral larval metamorphosis *in vitro* were closely related TBP-producing Pseudoalteromonads (GBR: 2001, 2011^3^,^4^; Caribbean: 2014^12^, Okinawa: 2014 ^this study^). Although these bacteria produce several other bioactive metabolites^30^, TBP was the only metabolite that induced larval metamorphosis. As previously demonstrated for larvae of a dominant reef-building coral of the GBR, we found that bacterial densities of at least 7,000 TBP-producing Pseudoalteromonads per mm^2^ were required to elicit statistically significant levels of larval metamorphosis^4^. Here we report the quantification of total epiphytic Pseudoalteromonads (<3 cells mm^−2^) and of TBP-producing Pseudoalteromonads (<1 cell mm^−2^) on two species of settlement-inducing CCA for the first time (Supplementary Table S1). While highly variable in abundance, these bacterial densities were orders of magnitude below the threshold density required to elicit coral larval metamorphosis, as previously reported from cultured biofilms^4^. Furthermore, chemical analyses of live CCA surfaces, settlement-inducing extracts and chromatographic fractions derived from CCA by NMR, LC-MS, DESI-MS and ESI-FTMS, did not detect TBP. This is despite TBP being readily detectable by ESI-MS five orders of magnitude below metamorphosis-inducing threshold concentrations.

For successful recruitment, coral larvae need to permanently attach to a benthic surface, a behaviour commonly observed in the presence of live CCA^21^,^25^^, this study^, coral reef biofilms^28^, and organic extracts and fractions of CCA^21^^, this study^ (Table 1). However, larvae of most coral species under investigation did not attach during metamorphosis when exposed to TPB and TBP-producing Pseudoalteromonads^3^,^4^^, this study^ (Figs. 1 & 2). The only exception observed in this study were larvae belonging to two species of the family faviidae: *L. pupurea,* a brooder and *F. pallida* a spawner. In these two species, the numbers of unattached polyps were not different from the control. However, when a phylogenetically diverse spectrum of larvae obtained from spawning acroporidae, merulinidae, poritidae and siderastreidae was tested in response to TBP^3^,^4^^, this study^, consistently a certain fraction of larvae did not attach. This observation was consistent across our locations (Great Barrier Reef and Ningaloo Reef, Singapore, Guam, Okinawa) and notably also observed in the Caribbean^12^.

The widespread and frequent failure of corals to attach to a substratum as part of the morphogenetic response, together with the lack of detection of TBP and the very low numbers of TBP-producing Pseudoalteromonads in field samples, cast doubt on the ability of this bacterial metabolite to drive successful recruitment in the field. In further support of the unusual response of coral larvae to TBP, pre-competent coral larvae of *A. millepora* and *A. tenuis* (1 – 4 days old) were consistently rendered immobile or died when exposed to TBP (see Fig. 2). Notably, this did not occur in pre-competent larvae exposed to live CCA where larvae continued swimming, developed normally and eventually settled and metamorphosed (Table 1).

The effect of TBP on both pre-competent and competent coral larval behavior is reminiscent of a range of neuroactive signaling compounds, for example GLW-amide neuropeptide, that elicit similar effects on coral or other invertebrate larvae^14^,^15^,^16^,^17^,^18^ (see Fig. 2). A range of coral larval genes encoding neurotransmitter functions were significantly up-regulated in the presence of TBP^31^ and one of these genes was significantly regulated in response to GLW-amide neuropeptide (Supplementary Table S5). In comparison, none of these neurotransmitter genes showed significant up- or down-regulation in response to settlement-inducing CCA cues^32^ (Supplementary Table S5). While the mechanism of action of TBP is unclear and the role of TBP as a neuroactive signalling compound is speculative, it is clear that there are profound differences in the response of coral larvae to TBP when compared to the response to CCA. These results suggest that TBP and TBP-producing bacteria are not the causative agents of coral larval settlement and metamorphosis on CCA.

To test if other, possibly uncultivable epiphytic bacteria on live or dead CCA contributed to the induction of larval settlement of *A. millepora* and *A. tenius* by CCA, the viability of epiphytic bacteria on CCA was either chemically (antibiotics, organic solvents) or physically (heat) moderated or eliminated altogether. After most of these treatments, CCA particulates still elicited the same high percentage of larval settlement and metamorphosis as untreated controls (Fig. 3, Table 1), an observation consistent with a similar study^21^ and strongly implying that larval settlement of these acroporid corals in response to CCA was not due to a viable bacterial source.

We thus investigated other settlement cues associated with CCA *per se*, for which there is also some prior evidence^25^. Various experimental treatments (Fig. 3) released settlement cues from CCA. The hot aqueous extraction of CCA under pressure released a water soluble settlement cue of high molecular weight (>100 kDa) that, unlike TBP and TBP-producing bacteria, caused exclusively larval settlement and metamorphosis (Table 1). The analysis of this extract revealed ^1^H-NMR signals consistent with those of high molecular polysaccharides. The hydrolysis of this extract under conditions that typically cleave oligomeric sugars deactivated its inductive property, suggesting that this cue was an intact polysaccharide or that the extract contained other component(s) susceptible to hydrolysis. It is therefore likely that matrix polysaccharides of CCA, mainly sulfated galactans^33^,^34^ released upon hot extraction, represent the high molecular weight settlement cue from CCA, which is accordance with prior findings^19^.

In addition to this high molecular weight settlement and metamorphosis cue, organic extraction of CCA revealed other cues of low molecular weight (Fig. 3). All of these cues induced the same level of larval settlement and metamorphosis as live CCA (Table 1). While previous studies have reported the presence of coral larval settlement cues in organic CCA extracts^20^,^21^, we demonstrate here for the first time that bioassay-guided purification confined these cues to chromatographic fractions associated with glycoglycerolipids (Supplementary Fig. S3). The predominant compounds in the bioactive fractions were fully elucidated by NMR and ESI-FTMS as the monoacylated glycoglycerolipids (2*S*)-1-*O*-(7*Z*,10*Z*,13*Z*-hexadecatrienoyl)-3-*O*-β-D-galactopyranosyl-*sn*-glycerol (MGMG; (**1),** Supplementary Fig. S2) and (2*R*)-1-*O*-(palmitoyl)-3-*O*-α-D-(6’-sulfoquinovosyl)-*sn*-glycerol (SQMG; (**2**), Supplementary Fig. S2). While the two bioactive TLC fractions containing MGMG and SQMG were spectroscopically (^1^H-NMR) pure, they contained an impurity that was below NMR detection levels but visible as a blue fluorescent halo under 365 nm. When this co-eluting compound were separated by further chromatography, neither the pure glycoglycerolipids MGMG and SQMG, nor the unidentified co-eluting substance, nor the combined sample trace across the entire TLC plate containing all former sample constituents returned a settlement cue. This loss of bioactivity in the final purification was highly reproducible across different chromatographic systems, including HPLC and could not be explained by a concentration-dependent effect. Possibly MGMG and SQMG may form bioactive conformational units with one or several unidentified minor compounds in the extract which are irretrievably cleaved in the final purification step. This notion is reminiscent of lipid rafts, structured aggregations of amphipathic lipid micro domains in biological cell membranes that irreversibly separate during chromatography^35^. An alternate possibility for the loss of activity is that a minor compound that complements the inductive effect of a sample fraction was unstable and degraded upon isolation, rendering the entire sample fraction inactive to larval settlement.

When exploring the suitability of benthic substratum coral larvae are affected by a combination of environmental variables, such as chemical cues, light, color and surface rugosity^9^,^10^,^11^. Natural reefal microbial communities can also play a role in triggering larval settlement and metamorphosis, but thus far conclusive evidence that bacteria, such as *Pseudoalteromonas* sp. are important to coral recruitment *in situ* remains elusive. This study posits that at least two purified, chemically different fractions derived from CCA represent ecologically plausible cues for larval settlement and metamorphosis of hard corals. One of these fractions was dominated by a high molecular weight compound, presumably a large polysaccharide fragment released from the cell wall matrix of CCA upon hot extraction, the other was associated with monoacylated glycoglycerolipids. Glycoglycerolipids occur widely in marine algae and cyanobacteria (reviewed in^36^) and glycoglycerolipid-containing fractions have previously been reported as larval settlement cues for sea urchins^37^ and jellyfish^38^. Hence, this compound class may indeed have wider implications for the recruitment of benthic marine invertebrates.

For both classes of compounds, we showed that they could be isolated directly from live CCA and were chemically detectable in all active fractions, unlike TBP which was isolated from a cultured bacterium (*ex situ*) and not detected in natural samples. In addition we showed that the purified fractions induced i) identical behavioral responses of larvae as per live CCA at all larval developmental stages, ii) larval settlement and metamorphosis in response to equivalent concentrations as present (volumetrically) in CCA, and (iii) larval settlement and metamorphosis in large non-confined test vessels under flow-through conditions (Table 1). Although we do not yet know the concentrations of these compounds on the surface of CCA, their presence in the cell walls and membranes^36^ most likely exposed by constant sloughing^20^ supports the plausibility of their detection by larvae *in situ*.

By providing a chemical signature of strong larval settlement and metamorphosis cues, CCA likely play a fundamental ecological role in the fine-scale recruitment of many coral species. CCA are key reef-building species that cement loose coral rubble with a hard carbonate skeleton and thus provide a suitable site for attachment of corals. In support of their ecological role as keystone species for coral reefs, CCA predominantly occur in the photic zone of oligotrophic waters, thus in ideal habitat conditions for many reef-building corals^20^,^32^. These factors may have favored the evolution of coral larval settlement to CCA in high-light oligotrophic rather than eutrophic waters, where fast-growing dominant turf algae impede coral recruitment^32^.

Our understanding of the chemical ecology of larval settlement and surface colonization in marine benthic systems, particularly for positive inducers of settlement, is far less advanced compared to other chemically mediated strategies of marine organisms in particular plant-herbivore or predator-prey interactions^39^ and deterrents of settlement^40^. While the “ecological realism” of plant herbivore and predator prey interactions has been a focus for two decades or more, the field of marine larval settlement has primarily focused on identifying stimulants of larval settlement without an equivalent focus on the ecological context of these cues; thus there still remain very few examples where analogous criteria of ecological plausibility has been employed for settlement inducers (e.g.^8^,^41^,^42^,^43^).

In conclusion, here we have provided a critical assessment of diverse settlement cues derived from the CCA holobiont, and their ecological context, for several families of globally important reef-building corals across a wide geographical range. Our results challenge the recent emphasis on bacterially-derived cues as the main inducers of coral larval settlement, suggesting instead an important role for algal-derived cues. Future studies focusing on the *in situ* presentation and quantification of these cues, and resulting larval responses, will further our understanding of the widespread potential of CCA to steer larval recruitment in marine reef ecosystems.

## Material and Methods

### Ethics statement

The work at various sites including collection of coral colonies was carried out with permits by the relevant authorities: Great Barrier Reef (GBR) G09/30237.1, G10/33440.1, G12/35236.1 (GBR Marine Park Authority); Ningaloo Reef Marine Park CE002767, SF007369 (Western Australia Department of Environment and Conservation); Singapore M4060980 (Singapore National Parks Board); Okinawa 18-16, 19-16 (Okinawa Prefectural Ordinance); Guam vn2001 (Department of Aquatic and Wildlife Resources). All colonies were returned to the reef within a week after spawning and reattached to minimize damage to the reef. No ethical approval was required for any of the experimental research described herein.

### Study organisms, sample locations and assay procedures

This study was conducted from 2009 - 2013 in five geographic regions: East Australia (Great Barrier Reef, herein referred to as GBR), Western Australia (Ningaloo Reef, herein referred to as WA), Singapore, Guam and Japan (Okinawa) with broadcast spawning acroporidae, siderastreidae, favidae and brooding favidae (GBR and WA: *Acropora millepora*, *A. tenuis*; Singapore: *A. millepora*; Guam: *A. globiceps*, *A. surculosa*, and *Leptastrea purpurea*; Okinawa: *A. humilis, A. tenuis*, *A. intermedia, Montipora hispida, Favia pallida, Pseudosiderastrea tayamai*). The experimental methods employed across different geographic locations, time points and coral species differed slightly. The entire set of experiments was not conducted at all locations. Where indicated, the broad scale approach was used to directly cross compare larval behavioural responses between species across different locations, but this was not possible at each site. For clarity we provide the reader with all data of coral larval responses to different cues. Notably, the effects of TBP on larval settlement and metamorphosis were tested with synthetic TBP in the GBR, WA, Singapore and Guam. TBP was isolated from bacterial cultures and tested as such in the GBR and Okinawa. TBP-producing Pseudoalteromonads were quantified at two time points in the GBR. The chemical inactivation and isolation of chemical cues from CCA has been performed and tested in the GBR, WA, Singapore and Guam. Larval settlement assays with competent larvae were replicated (n = 6 - 10) in sterile 12-well plates (Australia, Singapore) or 10 mL glass petri dishes (Guam, Okinawa) containing 0.2 μm-filtered seawater (FSW) at 27–28 °C and no more than one larva mL^−1^. Extracts, fractions and isolated compounds were dissolved in appropriate solvents, evaporated in the test vessels, filled with FSW and tested in a logarithmic serial dilution against controls of FSW. In the presence of CCA these corals consistently settle and metamorphose into attached polyps. The CCA *Porolithon onkodes*, *Hydrolithon reinboldii and Neogoniolithon fosliei* were collected from the same locations as coral brood stocks. Coral collection, spawning, larval culture and larval assay techniques were adopted from published procedures^3^,^4^,^21^,^31^.

Tetrabromopyrrole (TBP) was synthesized according to Palmer^44^ and tested synthetically (GBR, WA, Singapore and Guam) or isolated from bacterial cultures (as per^4^) and tested (GBR and Okinawa). All TBP samples were purified by chromatography (as per^4^). TBP is unstable^44^,^45^, especially as a solid, therefore dose-response curves of TBP can be inaccurate. Identical TBP aliquots were stored or shipped in the dark at −20 °C to ensure comparability of experiments at the different test sites. After 6, 8 or 24 hours larval responses were observed under the dissecting microscope. The larval responses to settlement cues were evaluated depending on whether larvae firmly attached to the substratum and/or whether they underwent morphological changes from a planula larva to a polyp. Larval metamorphosis was recorded once larvae had developed into disc-shaped structures with pronounced flattening of the oral-aboral axis and typically had obvious septal mesenteries radiating from the central mouth region (polyps)^21^. The following categories of larval behavior were observed and scored in the assays: (i) larval settlement and metamorphosis (with attachment), (ii) larval metamorphosis without attachment (floating polyps), (iii) swimming larvae (no response to settlement cues).

### Larval settlement bioassay-guided screening of bacterial isolates

The isolation, culture and identification of TBP-producing *Pseudoalteromonas* strains J010 and J021 has been described previously^4^. A similar screening was performed with 3 month-old marine biofilms on submerged terracotta tiles in a reef off Aka Island, Okinawa, Japan (26°11’N, 127°17’E). In total, 160 bacterial isolates were cultured on sterilized nitrocellulose filter placed on 1/10 diluted ZoBell2216E agar plates and screened with larvae of *A. tenuis*.

### Quantification of TBP-producing Pseudoalteromonads on CCA

The total abundance of epiphytic Pseudoalteromonads and of the two known TBP-producing *Pseudoalteromonas* strains (J010 and J021^4^) on the surface of *P. onkodes* and *N. fosliei* was determined by real-time quantitative PCR (q-PCR). Each species was sampled in the GBR with replication (n = 6) in October and December 2010. Total Pseudoalteromonads were quantified with the general bacterial primer Eub341F^46^ and *Pseudoalteromonas*-specific primer Psalt815R^47^. To selectively target *Pseudoalteromonas* strains J010 and J021, specific primers Ps-F1 and Ps-R6 were designed based on the bacterial ITS region (the detailed q-PCR procedures can be found in Supplementary Materials and Methods online).

### Bioassay-guided treatments of CCA

The procedures of bioassay-guided treatments (Fig. 3) are given in Supplementary Materials and Methods.

### Larval settlement assays with embedded CCA-derived cues

Larval settlement cues obtained by ultrafiltration (>100 kDa, Fig. 3) and VFC (pooled fractions 8 and 9, see Supplementary Fig. S3) of CCA extracts were embedded in resins (paraffin and carrageenan, n = 15) at the equivalent volumetric concentration as in CCA (Supplementary Materials and Methods). The embedded cues were tested under flow-through conditions (ca. 1 L per min) in 500 L vessels (n = 3) at a low ratio of cue-to-water volume together with controls (resins without cues). Statistical analysis of larval settlement and metamorphosis in the tank assay was done by one-way ANOVA followed by Tukey’s pairwise comparison. Because of the scale of the tank relative to the scale of the larvae, and the fact that cues were affixed to the tiles and only detectable on contact by larvae, we considered different treatments (tile types) and replicate tiles to be independent. Moreover, the number of larvae in the tank was high (ca. 0.5 per ml in a 500 L tank) relative to the total number settled (<100), making it unlikely that settlement on one tile type restricted the number of larvae settling on other types.

### Statistical analysis and data treatment

Since data did not fulfil the conditions of normality and homoscedasticity, and could not be improved by transformation, they were analyzed by non-parametric Kruskal–Wallis one-way ANOVA on ranks. Further, comparisons were made between treatments and controls by permutational analysis of variance in the PERMANOVA routine of PRIMER v6^48^. PERMANOVA with 999 permutations based on Euclidean distance followed by pairwise comparisons was used to statistically evaluate experimental treatments.

## Additional Information

**How to cite this article**: Tebben, J. *et al.* Chemical mediation of coral larval settlement by crustose coralline algae. *Sci. Rep.* **5**, 10803; doi: 10.1038/srep10803 (2015).

## Supplementary Material

Supplementary Information

## Figures and Tables

**Table 1:** Comparison of larval responses triggered by (i) live CCA and CCA extracts, (ii) the bacterium *Pseudoalteromonas* and its metabolite TBP and (iii) the neurotransmitter GLW-amide

	**CCA**			**Bacteria**		**Neurotransmitter**
**Larval behavior**	**Live**	**Organic extract (Glyco-glycerolipid)#**	**Aqueous extract (Polysaccharide)#**	***Pseudoaltero monas*** *	TBP*	**GLW-amide#**
Settlement and metamorphosis with attachment	>70%	>70%	>70%	<50%	<50%	<40%
Metamorphosis without attachment	0	0	0	>50%	>50%	>60%
Adverse effect on pre-competent larvae	No	No	No	Yes	Yes	Yes
Regulation of neurotransmitter genes (Table S5)	No	No	No	Yes	Yes	Partially
Detected in natural samples	n/a	Yes	Yes	No	No	n/a

^*^Acropora millepora, A. tenuis, A. globiceps, A. surculosa, Leptastrea purpurea, A. humilis, A. intermedia, A. digitifera, Montipora hispida, Favia pallida and Pseudosiderastrea tayamai in GBR, WA, Singapore, Guam and Okinawa; and # A. millepora and A. tenuis in GBR, WA and Singapore.
