# Supplementary material for: Chemical mediation of coral larval settlement by crustose coralline algae
Source: Sci Rep. 2015 Jun 4;5:10803. doi: 10.1038/srep10803 (PMC4650656; doi:10.1038/srep10803)
Supplement: Supplementary Information [file srep10803-s1.pdf]

TITLE

Chemical mediation of coral larval settlement by crustose coralline algae

SHORT TITLE

Coral larval settlement cues

Tebben J[1], Motti CA[2], Siboni N[2,3], Tapiolas DM[2], Negri AP[2], Schupp PJ[4], Kitamura M[5], Hatta M[6], Steinberg PD[1,7,8], Harder T[1]

Supplementary Information

SI Figures

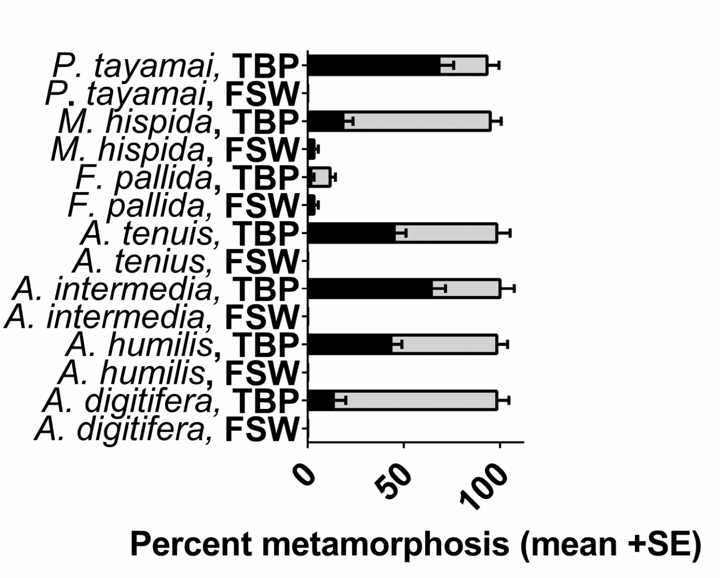

Figure S1: Larval response (+ SE, n = 6 - 10) to bacterially derived and purified TBP after 8 h. Seven coral species (*A. humilis, A. intermedia, A. digitifera, A. tenuis, Montipora hispida, Favia pallida* and *Pseudosiderastrea tayamai)* from Okinawa, Japan were used in larval settlement assays with bacterially derived TBP together with controls of filtered seawater (FSW). Black bars: mean percentage of larval metamorphosis without attachment, Grey bars: mean percentage of larval settlement and metamorphosis (with attachment).

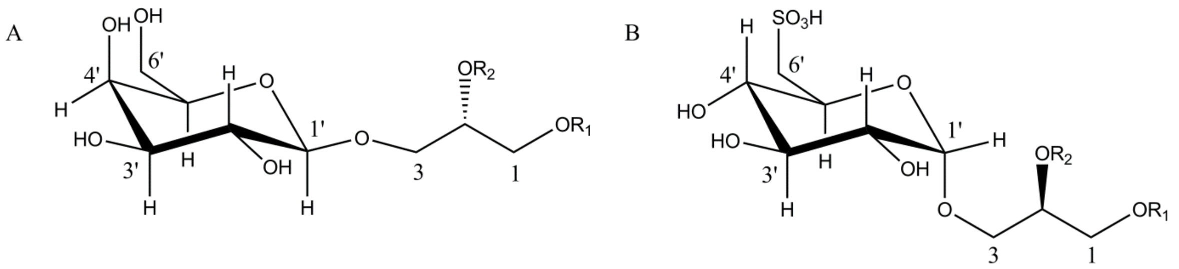

Figure S2: Chemical structures of A: MGDG ($R_1 = R_2$ = acyl side chain), MGMG (**1**, R_1 = acyl side chain, R_2 = H) and B: SQDG ($R_1 = R_2$ = acyl $C_{16:0}$ side chain) and SQMG (**2**, R_1 = acyl $C_{16:0}$ side chain, R_2 = H).

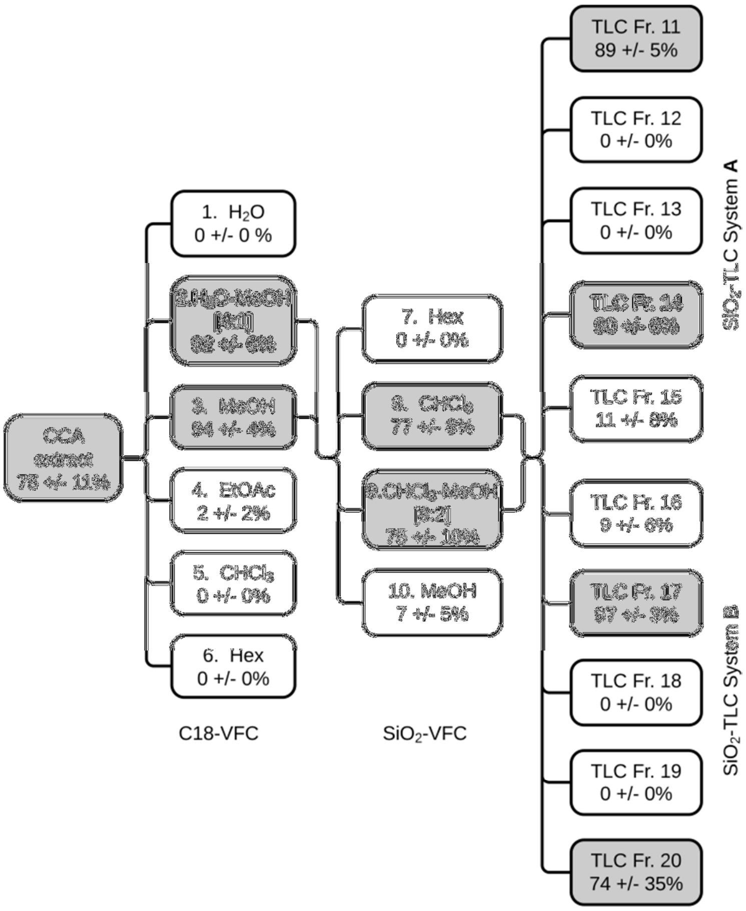

Figure S3: Bioassay-guided purification scheme of the organic extract of the crustose coralline alga (CCA) *Porolithon onkodes.* Numbered fractions were obtained by vacuum flash chromatography (VFC) and two thin-layer chromatography (TLC) protocols (SI Material and Methods). Bold squares represent fractions that (i) induced significant larval settlement compared to the negative control of filtered seawater (FSW; $p < 0.05$, pairwise PERMANOVA), and (ii) statistically the same response to live, untreated CCA ($\alpha = 0.05$, pairwise PERMANOVA) after 12 h ($n = 6$). Percentage values represent the mean larval settlement

response (± SE, n = 6) of *Acropora millepora* to these fractions after 12 h. Fraction 14 and 17 contained ^{1}H-NMR spectroscopically pure SQMG and MGMG. Fraction 11 and 20 contained chromatographically unresolved SQMG and MGMG.

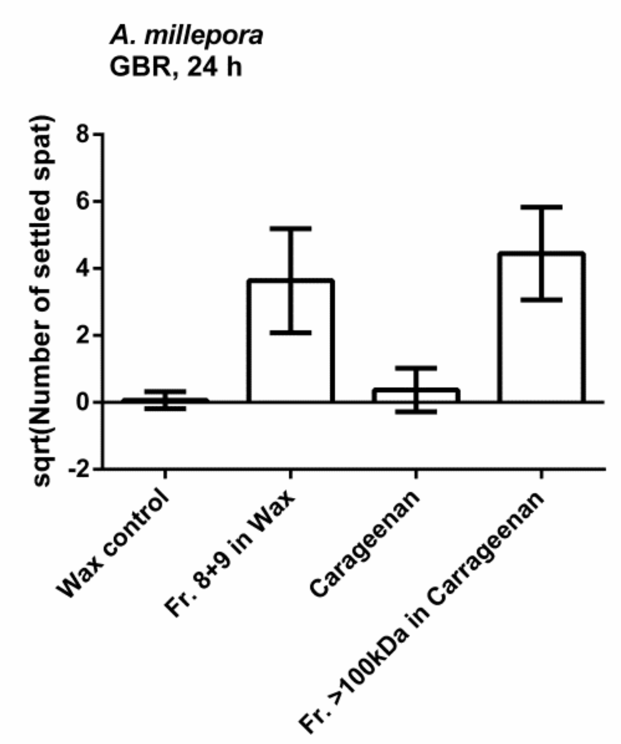

Figure S4: Larval settlement of *A. millepora* under flow-through conditions on cues obtained by ultrafiltration (Fr. >100kDa, Figure 3) and vacuum flash chromatography (Fr. 8+9, Figure S2) immobilized in paraffin (wax) and Carrageenan in comparison to controls. Bars show square root transformed mean of settled spat per replicate (25 cm^2, n = 15). Error bars are 95% confidence interval.

SI Tables

Table S1: Total abundance of epiphytic Pseudoalteromonads and of specific *Pseudoalteromonas* strains J010 and J021 on the surface of the crustose coralline algae *Porolithon onkodes* and *Neogoniolithon fosliei* sampled in the GBR determined by real-time quantitative PCR.

Crustose coralline alga (month/year)	Total Pseudoalteromonads (mean ± SE cm^{-2})	*Pseudoalteromonas* strains J010 and J021 (mean ± SE cm^{-2})
Neogoniolithon fosliei (09/2010)	1009 ± 698	10 ± 7
Neogoniolithon fosliei (12/2010)	2707 ± 2113	56 ± 25
Porolithon onkodes (09/2010)	2170 ± 1233	24 ± 5
Porolithon onkodes (12/2010)	1260 ± 1084	2 ± 1

Table S2: ^{13}C-NMR (150 MHz) shifts of glycoglycerolipids MGMG (**1**) and SQMG (**2**)

	MGMG (compound 1)[a]	MGMG [1 a]	SQMG (compound 2)[b]	SQMG [2 b]
**No.**	**δ_C, mult.**	**δ_C, mult.**	**δ_C, mult.**	**δ_C, mult.**
**1**	66.2, CH_2	66.6, CH_2	64.7, CH_2	66.4, CH_2
**2**	69.3, CH	69.7, CH	67.8, CH	69.2, CH
**3**	71.4, CH_2	71.9, CH_2	68.4, CH_2	70.0, CH_2
**1'**	104.9, CH	105.3, CH	98.0, CH	99.6, CH
**2'**	72.1, CH	72.6, CH	71.1, CH	72.5, CH
**3'**	74.5, CH	74.9, CH	72.7, CH	73.9, CH
**4'**	69.9, CH	70.3, CH	72.4, CH	73.5, CH
**5'**	76.4, CH	76.8, CH	67.7, CH	69.2, CH
**6'**	62.0, CH_2	62.5, CH_2	52.1, CH_2	53.3, CH_2
**1''**	175.3, qC	175.5, qC	ND, qC	176.1, qC
**2''**	34.6, CH_2	33.9, CH_2	33.6, CH_2	34.9, CH_2
**3''**	25.6, CH_2	25.9, CH_2	24.4, CH_2	25.7, CH_2
**4''**	30.2, CH_2	29.87, CH_2	28.9, CH_2	30.5, CH_2
**5''**	30.2-32.7, CH_2	30.3, CH_2	Envelope not resolved	30.5, CH_2
**6''**	27.3, CH_2	28.0, CH_2		30.5, CH_2
**7''**	128.8, CH	128.2, CH		31.0, CH_2
**8''**	129.5, CH	129.0, CH		31.0, CH_2
**9''**	26.3, CH_2	26.5, CH_2		31.0, CH_2
**10''**	130.4, CH	129.2, CH		31.0, CH_2
**11''**	132.6, CH	129.3, CH		31.0, CH_2
**12''**	26.3, CH_2	26.5, CH_2		31.0, CH_2

**13''**	128.8, CH	130.9, CH		30.3
**14''**	128.8, CH	132.8, CH	28.9, CH_2	32.9, CH_2
**15''**	27.9, CH_2	21.5, CH_2	22.2, CH_2	23.6, CH_2
**16''**	14.2, CH_3	14.6, CH_3	13.5, CH_3	14.8, CH_3

[a] MGMG in CD_3OD
[b] SQMG in $CD_3OD/CDCl_3$

Table S3: ^{1}H-NMR (600 MHz) shifts of glycoglycerolipids MGMG (**1**) and SQMG (**2**)

	**MGMG (compound 1)**[a]	**MGMG** [1 a]	**SQMG (compound 2)**[b]	**SQMG** [2 b]
**No.**	**δ_H (*J* Hz)**	**δ_H (*J* Hz)**	**δ_H (*J* Hz)**	**δ_H (*J* Hz)**
**1α**<br>**1β**	4.15, d (6.0)[d]<br>4.16, d (4.9)	4.13, dd (11.4, 5.9)<br>4.16, dd (11.4, 4.6)	4.07, dd (11.1, 6.1)<br>4.16, dd (10.9, 3.3)	3.96, dd (10.7, 6.3)<br>4.03, dd (10.7, 4.4)
**2**	4.00, dd (5.7, 4.8)	3.98, m	4.03, m	3.91, m
**3α**<br>**3β**	3.67, dd (10.5, 4.4)<br>3.92, dd (10.5, 5.1)	3.65, dd (10.5, 4.6)<br>3.90, dd (10.5, 5.2)	3.36, dd (10.1, 7.2)<br>3.93, dd (10.3, 2.3)	3.33, dd (10.7, 6.4)<br>3.71, dd (10.7, 4.4)
**1'**	4.24, d (7.6)	4.22, d (7.6)	4.78, d (3.3)	4.59, d, (3.4)
**2'**	3.55, dd (9.8, 7.7)	3.53, dd (9.7, 7.6)	3.45, dd (9.5, 3.1)	3.18, dd (9.3, 3.4)
**3'**	3.49, dd (9.7, 3.3)	3.46, dd (9.7, 3.4)	3.63, m	3.40, dd (9.3, 9.3)
**4'**	3.85, br d (3.3)	3.82, dd (3.3, 0.9)	3.18, br t (9.0)	2.96, dd (9.3, 8.8)
**5'**	3.53, m	3.51, m	3.99, br t (9.0)	3.88, ddd (8.8, 5.9, 4.9)
**6'α**<br>**6'β**	3.74, br t (5.7)	3.71, dd (11.3, 5.3)<br>3.76, dd (11.5, 6.9)	2.99, dd (14.5, 7.4)<br>3.36, dd (10.1, 7.2)	2.61, dd (13.7, 5.9)<br>2.91, dd (13.7, 4.9)
**2''**	2.36, dt (10.6, 7.5)	2.35, t (7.4)	2.31, t (7.6)	
**3''α**<br>**3''β**	1.69, ddd (12.0, 10.3, 6.6)<br>1.62, m	1.62, m	1.57, dt (14.9, 7.4)	
**4''**	1.31-1.37, m	1.37, m	1.25, m (envelope)	
**5''**	1.31-1.37, m	1.37, m	1.25, m (envelope)	

**6’’**	2.13, m	2.09, m	1.25, m (envelope)	
**7’’**	5.35-5.39, m	5.34, m	1.25, m (envelope)	
**8’’**	5.35-5.39, m	5.34, m	1.25, m (envelope)	
**9’’**	2.84, ddd (12.4, 10.4, 5.5)	2.80, br t (5.8)	1.25, m (envelope)	
**10’’**	5.35-5.39, m	5.34, m	1.25, m (envelope)	
**11’’**	5.35-5.39, m	5.34,m	1.25, m (envelope)	
**12’’**	2.84, ddd (12.4, 10.4, 5.5)	2.80, br t (5.8)	1.25, m (envelope)	
**13’’**	5.35-5.39, m	5.34, m	1.25, m (envelope)	
**14’’**	5.35-5.39, m	5.34, m	1.57, m	
**15’’**	2.08, m	2.09, m	1.25, m	
**16’’**	0.98, t (7.6)	0.97, t (7.6)	0.83, t (7.0)	

[a] MGMG in CD_3OD
[b] SQMG in $CD_3OD/CDCl_3$
[c] SQMG in DMSO-d_6/D_2O. Note that only the glycosyl and glycerol protons were reported.
[d] 2nd order coupling

1. Kwon, O. & Danishefsky, S.J. Synthesis of asialo GM_1. New insights in the application of sulfonamidoglycosylation in oligosaccharide assembly: Subtle proximity effects in the stereochemical governance of glycosidation. *J. Am. Chem. Soc.* **120**, 1588-1599 (1998).
2. Takahashi, Y., Itoh, K., Ishii, M., Suzuki, M. & Itabashi, Y. Induction of larval settlement and metamorphosis of the sea urchin Strongylocentrotus intermedius by glycoglycerolipids from the green alga Ulvella lens. *Mar. Biol.* **140**, 763-771 (2002).

Table S4: Glycosyl composition analysis result of hot aqueous extract of *Porolithon onkodes*

Glycosyl residue	Mass (µg)	Weight %[a]
Ribose (Rib)	n.d.	-
Arabinose (Ara)	n.d.	-
Rhamnose (Rha)	n.d.	-
Fucose (Fuc))	n.d.	-
Xylose (Xyl)	0.0	1.1
Glucuronic Acid (GlcA)	n.d.	-
Galacturonic acid (GalA)	n.d.	-
Mannose (Man)	0.1	3.5

Galactose (Gal)	0.4	12.1
Glucose (Glc)	2.9	83.3
N-Acetyl Galactosamine (GalNAc)	n.d.	-
N-Acetyl Glucosamine (GlcNAc)	n.d.	-
N-Acetyl Mannosamine (ManNAc)	n.d.	-
3-Deoxy-d-manno-Octulosonic Acid (Kdo)	n.d.	-
Σ=	3.5	
Total carbohydrate % by weight	1.2	

[a] values are expressed as weight percentage of total carbohydrate

Table S5: **Differential gene expression following exposure to bacteria- and CCA-derived cues and GLW-amide neuropeptide.** Significant (*p<0.05*) change in gene regulation is given as the % difference compared to the control. Green (+) represents those genes that were up- or down regulated.

	**Change in gene regulation as % of control**						
	Previously published data, used with permission [1]						
Gene [a]	Extracts of *Porolithon Onkodes*		TBP				GLW-amide neuropeptide
Assay 1	Fr. 8+9 1 h	> 100 kDa 1 h	1 h	2 h	3h	12h	1 h
GABA/GAT-2			+8.7	+18.1	+52.1	+104	
GABA/GAT-3			+18.2		+41.8	+120	
GABA/unc-47						+213	
GABA/ine				+40.6	+36.2		-34.1

1. Siboni, N., Abrego, D., Motti, C.A., Tebben, J. & Harder, T. Gene expression patterns during the early stages of chemically induced larval metamorphosis and settlement of the coral Acropora millepora. *PLoS. ONE* **9**, e91082 (2014).

SI Results and Discussion

Elucidation of the high molecular settlement cue

^{1}H-NMR spectra of the lyophilized autoclaved extract, the <100 kDa fraction and the >100 kDa fraction are shown in the SI dataset. While both fractions clearly contain oligosaccharides/polysaccharides (characterized by resonances at 3-4.5 ppm and anomeric signals in the 4.5-5.5 ppm region) the chemical shifts between 3-5 ppm suggests different

saccharide composition while the broadening of these signals in the >100 kDa fraction indicates larger molecular weight. ^{1}H-NMR of the >100 kDa fraction acid hydrolysis product shows significant sharpening of the broad peaks indicating the formation of smaller oligosaccharides and monosaccharides, providing further evidence for polysaccharides.

These results were further verified by glycosyl composition analysis, performed as previously described [1]. Monosaccharide methyl glycosides of the high molecular weight sample (>100 kD, 300 µg) were prepared by methanolysis (1 M HCl in methanol at 80 °C for 16 hours), followed by re-*N*-acetylation with pyridine and acetic anhydride in methanol (for detection of amino sugars). The sample was then per-*O*-trimethylsilylated with Tri-Sil (Pierce; at 80 °C for 0.5 hours) and analyzed by combined gas chromatography mass spectrometry (GC-MS; performed on an Agilent 7890A GC interfaced to a 5975C MSD, using Agilent DB-1 30m × 0.25 mm ID fused silica capillary column). This indicated the presence of several monosaccharides including xylose, mannose, galactose and glucose (Table S4).

Elucidation of the low molecular weight settlement cue

The molecular formula of **1** was established as $C_{25}H_{42}O_9$ based on ESI-FTMS (*m/z* $[M+Na]^+$ 509.2724, calculated for $C_{25}H_{42}O_9Na^+$ 509.2721;five degrees of unsaturation) and its structure confirmed by ^{1}H- and ^{13}C-NMR (Table S2, Table S3). The ^{13}C-NMR signals at δ_C 104.9, 72.1, 74.5, 69.9, 76.4 and 62.0, and the coupling constants of 7.6 Hz ($J_{1'-2'}$) and 3.3 Hz ($J_{3'-4'}$) in the ^{1}H-NMR spectra indicated the presence of a β-galactopyranose [2]. The NMR of **1** closely resembled that of monogalactosyldiacylglycerol (MGDG) except for the signals on the glycerol moiety. The chemical shifts for the *sn*-2 carbon were observed upfield for both ^{13}C and ^{1}H (δ_C 69.3; δ_H 4.00, dd 5.7, 4.8) compared to MGDG (δ_C 71.5; δ_H 5.27, m), indicating the *sn*-2 carbon was not acylated [3]. The fatty acid composition of **1** was determined by NMR and (+)-ESI-FTMS as a C16 fatty acid. Further, the ^{1}H-NMR and ^{13}C-NMR analyses of **1** in comparison with MGDG and the long-range connectivities observed in the HMBC spectrum between the ester carbonyl (δ_C 175.3) and the *sn*-1 β proton (δ_H 4.16 ppm), confirmed the fatty acid side chain was attached at *sn*-1 on the glycerol moiety. The stereochemistry at *sn*-2 of the glycerol was determined as 2*S* based on the finding that the chemical shifts for H1α (δ_H 4.15) and H1β (δ_H 4.16) are very close and the coupling constant $J_{2,3\alpha}$ (4.4 Hz) is smaller than that for $J_{2,3\beta}$ (5.1 Hz) as well as comparison with literature values [2, 4]. The isomery of the two double bonds (δ_C 132.6, 130.4, 129.5 and 128.8) in the C16 fatty acid side chain was determined to be *cis* based on the chemical shifts of the adjacent doubly allylic methylene signals (δ_C 26.2, δ_H 2.84, ddd 12.4, 10.4, 5.5, 2H). As a result, the chemical structure of **1** was determined as (2*S*)-1-*O*-(7*Z*,10*Z*,13*Z*-hexadecatrienoyl)-3-*O*-β-D-galactopyranosyl-*sn*-glycerol (Figure S2, A).

The molecular formula of **2** was established as $C_{25}H_{48}O_{11}S$ by accurate mass measurement and its structure determined by ^{1}H- and ^{13}C-NMR (Table S2, Table S3). Mass spectral analyses revealed peaks corresponding to $[M-H]^-$ *m/z* 555.2894 (calculated for $C_{25}H_{47}O_{11}S^-$ 555.2845) and the pseudomolecular ion of the sodium salt $[M-H+2Na]^+$ *m/z* 601.2608 (calculated for $C_{25}H_{47}O_{11}SNa_2^+$ 601.2629), which is reminiscent of sulfonoglycolipids, and accounted for a sulfonic acid moiety (SO_3H). The ^{13}C-NMR signals at δ_C 98.0, 71.1, 72.7, 72.4, 67.7 and the upfield shift of C-6' (δ_C 52.1), as well as the relatively small coupling in the ^{1}H-NMR of 3.3 Hz ($J_{1'-2'}$) indicated the presence of an α-linked glycosyl moiety. The large vicinal coupling constants observed for $J_{2'-3'}$ (9.5 Hz), $J_{3'-4'}$ (9.0 Hz) and $J_{4'-5'}$ (9.0 Hz), confirmed the glucopyranosyl nature of the sugar moiety. The ^{1}H-NMR and ^{13}C-NMR spectra of

2 closely resembled those of sulfoquinovosyldiacylglycerol (SQDG) except for the signals on the glycerol moiety. The signals for *sn*-2 were shifted upfield for both ^{13}C and ^{1}H (δ_C 67.8; δ_H 4.03, m) as compared to SQDG (δ_C 71.5; δ_H 5.31, m), that as for **1** the *sn*-2 carbon was not acylated [3]. The fatty acid composition in **2** was determined by NMR (no double bond signals were observed between δ 128.0 to 133.0) and ESI-FTMS indicating the presence of a fully saturated C16 fatty acid (palmitic acid, $C_{16:0}$). Further, the ^{1}H-NMR and ^{13}C-NMR analyses of **2** in comparison with SQDG and the long range connectivities observed in the HMBC spectrum between the ester carbonyl (δ_C 175.7) and the *sn*-1_α proton (δ_H 4.16 ppm, CD_3OD), confirmed the fatty acid side chain was attached at position *sn*-1 on the glycerol moiety. The stereochemistry at *sn*-2 of the glycerol unit was determined as 2*R* based on the coupling constants 7.2 Hz ($J_{3\alpha\text{-}2}$) and 2.3 Hz ($J_{3\beta\text{-}2}$), the upfield shift of *sn*-1α (δ_H 4.07, dd 11.1, 6.1) and *sn*-1β (δ_H 4.16, dd 10.9, 3.3) and comparison with literature values [4]. As a result, the chemical structure of **2** was determined as (2*R*)-1-*O*-(palmitoyl)-3-*O*-α-D-(6'-sulfoquinovosyl)-*sn*-glycerol (Figure S2, B).

1. Merkle, R.K. & Poppe, I. in *Methods in Enzymology.* (ed. Lennarz, J.K. & Hart, G.W.) 1-15 (Academic Press, 1994).
2. Kwon, O. & Danishefsky, S.J. Synthesis of asialo GM_1. New insights in the application of sulfonamidoglycosylation in oligosaccharide assembly: Subtle proximity effects in the stereochemical governance of glycosidation. *J. Am. Chem. Soc.* **120**, 1588-1599 (1998).
3. Sassaki, G.L., Machado, M.J., Tischer, C.A., Gorin, P.A.J. & Iacomini, M. Glycosyldiacylglycerolipids from the lichen Dictyonema glabratum. *J. Nat. Prod.* **62**, 844-847 (1999).
4. Hiraga, Y., Shikano, T., Widianti, T. & Ohkata, K. Three new glycolipids with cytolytic activity from cultured marine dinoflagellate Heterocapsa circularisquama. *Nat. Prod. Res.* **22**, 649-657 (2008).

SI Materials and Methods

q-PCR quantification of TBP-producing Pseudoalteromonads on CCA

Genomic DNA was extracted from six replicate CCA samples (total of 24 samples of 1.36 cm^2 each) extraction with the PowerSoil DNA Isolation Kit (Mo Bio Laboratories Inc., Solana Beach, CA). Total Pseudoalteromonads were quantified with the general bacterial primer Eub341F (5'-CCTACGGGAGGCAGCAG-3') [1] and *Pseudoalteromonas*-specific primer Psalt815R (5'-CCAGCTTCTAGTAGACATCGTT-3') [2] producing a product of 496 bp. To selectively target *Pseudoalteromonas* strains J010 and J021 specific primers Ps-F1 (5'-GATGATTGTTGCTTGGCCTGATG-3') and Ps-R6 (5'-CCTCCAACTGCCAAGGCATCC-3') were designed on the bacterial ITS region producing a product of 276 bp. The qPCR was performed on a RotoGene 300 (Corbett Research). A q-PCR calibration curve for *Pseudoalteromonas* strain J010 was constructed and included in each PCR run. The overnight culture in marine broth at 28°C was collected in five 1 mL aliquots after three cycles of washing with PBS followed by 10 min centrifugation (5200xg) at 10 °C. Four samples were used for DNA extraction (Mo Bio Laboratories Inc., USA) and one for viable count. The viable count of *Pseudoalteromonas* strain J010 on marine agar was $1.7 \pm 0.8 \times 10^8$ bacteria mL^{-1} genomic DNA (n = 4). PCR reaction mixtures included 10 µL of SensiMix SYBR No-ROX kit (containing 3 mM $MgCl_2$, SYBR Green I dye and dNTPs; Bioline, Australia), 0.5 µM each of the forward and reverse primers, 5 µL of the sample DNA and water to bring the total volume to 20 µL. All reactions were conducted on a RotoGene 300 (Corbett Research) real-time analyzer with the following cycling parameters: 95 °C for 10 min (polymerase activation), followed by 45 cycles

of 95 °C for 30/15 s (denaturation), 62 °C for 30/15 s (annealing) and 72 °C for 30/15 s (extension), for the total and specific primers, respectively. SYBR Green fluorescence was acquired at the extension step of each round (Results see Table S1). The Multiplex RT-qPCR measuring the gene expression of *A. millepora* in response to GLW-amide was done as previously described [3].

Bioassay-guided treatments of CCA

The live top layer of CCA was separated from the dead calcified material underneath using bone cutters and cleaned of macroscopic epibionts. Cleaned CCA were partitioned into three batches, the first was extracted (see below), the second autoclaved (see below), and the third was maintained alive in flow-through aquaria. Larval settlement assays with competent larvae were replicated (n = 6 - 10) in sterile 12-well plates (Australia, Singapore) or 10 mL glass petri dishes (Guam, Okinawa) containing 0.2 µm-filtered seawater (FSW) at 27 – 28 °C and no more than one larva mL^{-1}. Extracts, fractions and isolated compounds were dissolved in appropriate solvents, evaporated in the test vessels, filled with FSW and tested in a logarithmic serial dilution. Live *P. onkodes* specimens were treated with (i) antibiotics for 24 hours (1 mM chloramphenicol, 0.5 mM streptomycin and 2 mM kanamycin sulfate dissolved in FSW) or with (ii) 25 % household bleach in Milli-Q water or (iii) extracted (0.5 kg per 250 mL) with either hexane, ethylacetate, chloroform, methanol:chloroform [1:2], ethanol, methanol, or butanol, or (iv) extracted with hot or cold water. The hot aqueous extraction of CCA was done under autoclave conditions (121°C, 15 psi) using both, fresh and previously ethanol-extracted CCA. The cold aqueous extraction was done at room temperature. For all extractions, 0.5 kg of CCA (chipped into small pieces or ground by mortar and pestle) were extracted three times in 250 mL for 60 min. The aqueous extracts were then filtered, lyophilized, and redissolved in 50 mL Milli-Q water. Aliquots of these extracts (1 µL) were tested in a logarithmic concentration series from 10^3 to 10^{-6} with larvae of *A. millepora* and *A. tenuis* at the GBR site. The residual CCA powder was suspended in FSW and tested by transferring a small amount (~75 µL) into the settlement dish [4]. All other treatments (i-iii) were tested by placing CCA chips of ca. 5 mm^2 into the settlement dish with the red outer surface facing up [5, 6, 7]. The autoclaved aqueous extract (40 mL) was filtered through a 100 kDa membrane (Amicon). The filter residue on the membrane was then washed three times with 10 mL water and the aqueous filtrates pooled. The residue was further washed 3 times with 10 mL ethanol:water [9:1] after which the filter residue was recovered in 30 mL water. The pooled aqueous filtrate, the ethanol:water [9:1] filtrate and the filter residue were each dried and concentrated in 10 mL water. An aliquot of the 100 kDa-filter residue was hydrolyzed in 50 % trifluoroacetic acid at 100 °C for 1 h. The reaction mixture was dried and re-dissolved at the original concentration. All extracts, washings and filtrates were tested in the larval assays.

Chromatographic isolation of larval settlement cues from CCA extracts (Figure S2)

Live CCA (1 – 2.5 kg, top 5 mm layer chipped off the calcite skeleton / rubble) were extracted under sonication for 6 h. The pooled extract (ca. 5 L) was filtered (Whatman No. 1) and evaporated on 50 mL C18 chromatography resin. This material was loaded onto a pre-equilibrated flash chromatography column (C18-VFC, 80 x 600 mm) and serially eluted with 600 mL each of water (Fr.1), water:methanol [4:1] (Fr.2), methanol (Fr.3), ethylacetate (Fr.4), chloroform (Fr.5), and hexane (Fr.6). The pooled fractions Fr.2 and Fr.3 were evaporated on 50 mL Silica gel 60G. This material was loaded onto a pre-equilibrated flash column (SiO_2-VFC, 80

x 600 mm) and serially eluted with 600 mL each of hexane (Fr.7), chloroform (Fr.8), chloroform:methanol [8:2] (Fr.9), and methanol (Fr.10). Preliminary NMR analyses of fractions Fr.8 and Fr.9 revealed glycoglycerolipids as major components. The pooled fractions Fr.8 and Fr.9 were further separated by SiO_2-TLC with two mobile phase systems of different polarity and pH (A: chloroform:methanol [4:1]; B: chloroform:acetone:methanol:acetic acid:water [50:20:10:10:5]). TLC system A resulted in fractions Fr.11 – 15. TLC system B resulted in fractions Fr.16 – 20. Sample spots were visualized under 254 and 365 nm or by staining with iodine or orcinol/sulfuric acid. For the purification of compounds by TLC, samples were loaded across the baseline of several TLC plates and developed simultaneously. A one cm strip on each side of each plate was removed and stained. The R_f-values of the stained eluted spots were used as a guide to scrape fractions of interest from the unstained section of the same TLC plate. This technique allowed the use of destructive, compound class-specific stains to visualize lipid components whilst still yielding analytes at quantities sufficient for NMR analyses. Staining was done with ethanol-sulphuric acid (60:40, v/v) containing 0.4 % orcinol and heated to > 100 ºC [8]. The R_f-values of the stained eluted spots were used as a guide to scrape fractions of interest from the unstained section of the same TLC plate. TLC fractions were extracted with chloroform, chloroform:methanol [1:1] and methanol and the combined extracts concentrated under nitrogen. Any regions adjacent to stained spots were also scraped to test all compounds across the entire chromatogram. Fraction Fr.14 was re-chromatographed by TLC (chloroform:methanol:water [45:10:1] to yield **1**. Fraction Fr.17 was re-chromatographed by TLC (chloroform:acetone:acetic acid:methanol:water [45:15:10:10:5] to yield **2**.

General experimental and instrumental parameters

Ultrafiltration was achieved using 100 kDa membranes (Amicon). Preparative scale vacuum-flash chromatography (VFC) was performed in normal- and reversed-phase (Silica gel 60 G and Silica gel 60 RP-18, 40-63 um, Merck), referred to as SiO_2-VFC and C18-VFC. Partially purified fractions were analyzed by nuclear magnetic resonance (NMR) spectroscopy, liquid chromatography–mass spectrometry (LC-MS) and electrospray ionization Fourier transform-mass spectrometry (ESI-FTMS).

One- and two-dimensional NMR spectra were recorded in deuterated methanol (CD_3OD), chloroform ($CDCl_3$) and dimethylsulfoxide (DMSO-d_6; Cambridge Isotopes Laboratories Inc. USA) using 3 mm Bruker MATCH NMR tubes on a Bruker Avance 600 MHz NMR spectrometer equipped with cryoprobe. All NMR spectra were referenced to residual ^{1}H and ^{13}C resonances in deuterated solvents (CD_3OD, δ 3.31 for ^{1}H and δ 49.0 for ^{13}C; $CDCl_3$, δ 7.27 for ^{1}H and δ 77.0 for ^{13}C; DMSO-d_6, δ 2.50 for ^{1}H and δ 39.5 for ^{13}C). NMR spectra were recorded using standard Bruker pulse sequences. The low resolution mass spectra of crude extracts and fractions were measured either via direct injection or by LC-MS (Bruker Esquire 3000 quadrupole ion trap LC-mass spectrometer). Accurate high-resolution mass spectra were measured on a BrukerBioApex 47e FT-ICR mass spectrometer fitted with an Analytica of Branford ESI source (ESI-FTMS). For both low and high resolution MS ions were detected in either negative or positive mode within a mass range *m/z* 200-2000. Direct infusion of MS samples (0.2 mg mL^{-1}) was carried out using a Cole Palmer 74900 syringe pump at a flow rate of 150 $\mu L\ h^{-1}$. Compressed gases were supplied by BOC Gases and were at least 99.99% pure. Purified water was obtained from a Milli-Q water purification system (Millipore). All other solvents used were HPLC grade (Mallinckrodt). Monogalactose diacylglycerol (MGDG),

sulfoquinovosyl diacylglycerol (SQDG) and digalactose diacylglycerol (DGDG) were obtained from BioLipids. All other chemicals were sourced from Sigma-Aldrich.

Larval assays with immobilized CCA-derived larval settlement cues

The petri-dish assay format widely used to screen larval settlement cues can be prone to artefacts because larvae are exposed to cues in very small volumes of stagnant water, possibly confounding a real choice of larvae for the cue surface. To test the CCA-derived larval settlement cues under flow-through conditions in large vessels a low ratio of cue-to-water volume, similar to that of CCA was required. Larval settlement cues obtained by hot aqueous and organic extraction of CCA were purified by ultrafiltration (Figure 3) and vacuum flash chromatography (Figure S2), respectively, immobilized on solid substrates and tested under flow conditions (ca. 1 L per min) in 500 L seawater vessels containing 1 larva per mL. Briefly, aliquots of pooled column fractions Fr. 8 and 9 (Figure S2) and the >100 kDa ultrafiltration residue of the hot aqueous CCA extract (Figure 3) were immobilized in paraffin wax or 10% carrageenan, respectively. Extract from 50 cm^3 CCA was embedded in 50 cm^3 of wax and carrageenan and spread out on 25 cm^2 terracotta tiles (1-2 mm thickness). Each tank received five tiles of both cues and five tiles containing each immobilization matrix in a 4 x 5 randomized Latin square design. These assays were replicated (n = 3).

1. Muyzer, G., Dewaal, E.C. & Uitterlinden, A.G. Profiling of complex microbial populations by denaturing gradient gel-electrophoresis analysis of polymerase chain reaction-amplified genes coding for 16S ribosomal RNA. *Appl. Environ. Microb.* **59**, 695-700 (1993).
2. Skovhus, T.L., Ramsing, N.B., Holmstrom, C., Kjelleberg, S. & Dahllof, I. Real-time quantitative PCR for assessment of abundance of Pseudoalteromonas species in marine samples. *Appl. Environ. Microb.* **70**, 2373-2382 (2004).
3. Siboni, N., Abrego, D., Motti, C.A., Tebben, J. & Harder, T. Gene expression patterns during the early stages of chemically induced larval metamorphosis and settlement of the coral Acropora millepora. *PLoS. ONE* **9**, e91082 (2014).
4. Tebben J, Guest JR, Sin TM, Steinberg PD, Harder T. Corals Like It Waxed: Paraffin-Based Antifouling Technology Enhances Coral Spat Survival. *PLoS ONE* 2014, **9**(1)**:** e87545.
5. Harrington, L., Fabricius, K., De'Ath, G. & Negri, A. Recognition and selection of settlement substrata determine post-settlement survival in corals. *Ecology* **85,** 3428-3437 (2004).
6. Negri, A.P., Webster, N.S., Hill, R.T. & Heyward, A.J. Metamorphosis of broadcast spawning corals in response to bacteria isolated from crustose algae. *Mar. Ecol. Prog. Ser.* **223**, 121-131 (2001).
7. Tebben, J. et al. Induction of larval metamorphosis of the coral Acropora millepora by tetrabromopyrrole isolated from a Pseudoalteromonas bacterium. *PLoS. ONE* **6**, e19082 (2011).
8. Svennerholm, L. The quantitative estimation of cerebrosides in nervous tissue. *J. Neurochem.* **1**, 42-53 (1956).
